# Learning the Ropes Foundations: A pilot evaluation of a webinar for older adults at risk of dementia

**DOI:** 10.1002/alz70858_099779

**Published:** 2025-12-25

**Authors:** Keera N. Fishman, Linda Truong, Christopher Pilieci, Iris Yusupov, Gillian Rowe, Renee Climans, Kelly J Murphy

**Affiliations:** ^1^ Baycrest Hospital, Toronto, ON, Canada; ^2^ Ontario Shores Centre for Mental Health Sciences, Toronto, ON, Canada; ^3^ Kunin‐Lunenfeld Centre for Applied Research & Evaluation, Baycrest Academy for Research and Education, Toronto, ON, Canada; ^4^ Autism Wellness, Toronto, ON, Canada; ^5^ West Park Healthcare Centre, Toronto, ON, Canada; ^6^ University of Toronto, Toronto, ON, Canada; ^7^ Creative Solutions, Toronto, ON, Canada; ^8^ Baycrest, Toronto, ON, Canada; ^9^ Springboard Clinic, Toronto, ON, Canada

## Abstract

**Background:**

Mild cognitive impairment (MCI) affects 1 in 10 older adults and significantly increases risk of future dementia, a disease impacting over 63 million people worldwide. Despite the need for dementia prevention, resources to empower individuals with MCI and their families to manage cognitive challenges and promote brain health remain limited. The online **Learning the Ropes Foundations** webinar was developed to provide a free, accessible, evidence‐based resource to delay or prevent progression from MCI to dementia. The webinar is available in English, French, and Spanish, with closed captions, transcripts, and handouts to ensure accessibility for diverse populations.

**Methods:**

Between January and July 2024, approximately 1600 individuals viewed the Learning the Ropes Foundations webinar. Of these, 69 participants (42 identifying with MCI/cognitive decline and 27 family members/friends) completed a post‐webinar survey evaluating its clarity, usability, and content. The survey included 5 Likert‐scale questions regarding participants’ level of agreement with applying information from the webinar to their everyday lives and recommending the webinar, as well as their motivation to adopt behaviour strategies from the webinar. The survey also included 3 open‐ended questions regarding participants’ overall experience with the webinar. Data were analyzed using descriptive statistics and content analyses.

**Results:**

Of the respondents, 91% of individuals living with cognitive decline and 100% of family members/friends agreed they could apply the information to their everyday lives. Additionally, 93% of individuals with cognitive decline and 100% of family members/friends agreed they would recommend the webinar to others. Lastly, 91% of all participants were motivated to adopt at least one behaviour change related to using a memory organizer, participating in recreational activities, or practicing stress management techniques. Qualitative feedback emphasized the overall value of the webinar (e.g., “It gave me tips to help me hold on to my brain power.”).

**Conclusions:**

The Learning the Ropes Foundations webinar has strong potential as an accessible, evidence‐based resource for individuals living with cognitive decline and their families to manage cognitive challenges and promote brain health. The positive feedback demonstrates the webinar's potential as a scalable tool for improving quality of life, prolonging independence, and promoting aging in place.

